# Arsenate Retention by Epipsammic Biofilms Developed on Streambed Sediments: Influence of Phosphate

**DOI:** 10.1155/2013/591634

**Published:** 2013-09-24

**Authors:** D. M. Prieto, R. Devesa-Rey, D. A. Rubinos, F. Díaz-Fierros, M. T. Barral

**Affiliations:** ^1^Department of Soil Science and Agricultural Chemistry, Facultad de Farmacia, Campus Vida, 15782 Santiago de Compostela, Spain; ^2^Defense University Center, Escuela Naval Militar, Plaza de España 2, 36920 Marín, Spain

## Abstract

Natural geological conditions together with the impact of human activities could produce environmental problems due to high As concentrations. The aim of this study was to assess the role of epipsammic biofilm-sediment systems onto As (V) sorption and to evaluate the effect of the presence of equimolar P concentrations on As retention. A natural biofilm was grown on sediment samples in the laboratory, using river water as nutrient supplier. Sorption experiments with initial As concentrations 0, 5, 25, 50, 100, 250, and 500 **μ**g L^−1^ were performed. The average percentage of As sorbed was 78.9 ± 3.5 and 96.9 ± 6.6% for the sediment and biofilm-sediment systems, respectively. Phosphate decreased by 25% the As sorption capactity in the sediment devoid of biofilm, whereas no significant effect was observed in the systems with biofilm. Freundlich, Sips, and Toth models were the best to describe experimental data. The maximum As sorption capacity of the sediment and biofilm-sediment systems was, respectively, 6.6 and 6.8 **μ**g g^−1^ and 4.5 and 7.8 **μ**g g^−1^ in the presence of P. In conclusion, epipsammic biofilms play an important role in the environmental quality of river systems, increasing As retention by the system, especially in environments where both As and P occur simultaneously.

## 1. Introduction

Arsenic (As) is a ubiquitous contaminant which is widely distributed in the environment. Due to its toxicity, its presence in soils, sediments, and water, even at very low concentrations, may cause serious health hazards, increasing the incidence of cancer and dermatological, vascular, and cerebrovascular diseases. For this reason, it was one of the first chemicals recognized as carcinogens [1]. It is estimated that 40 million people worldwide are at risk from drinking As-contaminated water [2]. Several cases of people affected by As pollution have been reported; thus, for example, thousands of arsenic poisoned patients were identified in Bangladesh, suffering from skin lesions and gangrene in legs as well as various types of cancer [3]. Consequently, the World Health Organization (WHO) has set the level of arsenic allowed at 10 *μ*g L^−1^ in drinking water [4]. 

Environmental As problems are commonly the result of mobilization under natural conditions, such as weathering of arsenic-bearing minerals and geothermal sources, but human activities have contributed to an important additional impact by means of mining processes, fossil fuel combustion, and the use of arsenic in pesticides, herbicides, crop desiccants, and livestock feed [5]. 

Dissolved As can occur in aquatic systems in both organic and inorganic forms. Inorganic As species predominate in sediments and water, but, in contrast, organoarsenic compounds prevail in marine organisms [6]. 

The inorganic As can be present in natural aquatic systems in four oxidation states: +V (arsenate), +III (arsenite), 0 (elemental As), and −III (arsine). The oxidation state is determined by pH and Eh. As (V) and As (III) are the common valence states in natural waters. As (V) is the thermodynamically stable form that generally predominates in oxic surface waters, whereas As (III) is favoured in environments with low pH and low redox potential [7]. In natural waters and at normal pHs, arsenate and arsenite are present as oxyanions (such as H_2_AsO_4_^−^ and HAsO_4_^2−^) and as neutral aqueous species (H_3_AsO_3_), respectively [8].

As previously mentioned, As may also occur in organic forms due to biological transformation of inorganic arsenic species. In the literature, this fact has been widely reported, showing that microorganisms may methylate As species as monomethylarsonic acid (MMAA), dimethylarsinic acid (DMAA), and trimethylarsine oxide (TMAO) [9, 10]. Additionally, arsenosugars could be produced by seaweed [11], whereas arsenobetaine and arsenocholine could be produced by marine animals [12–14].

Arsenic toxicity is dependent on the chemical form in which As is presented (inorganic or organic) and on its oxidation state. Traditionally, the inorganic forms of As have been considered more toxic than the organic forms [15]. Among inorganic forms, As (III) is in general considered more toxic, soluble and mobile than As (V) [16]. 

In rivers, sediments act as a significant sink of As, although changes in the river flow or in other environmental conditions (Eh, pH, and changes in water composition) may cause adsorption or desorption processes which should be controlled. In the last years, studies based on As adsorption onto sediments were reported by Rubinos et al., Bostick et al., Stollenwerk et al., Borgnino et al., and Mandal et al. [17–21]. Arsenic adsorption capacity has been related to the content of metal oxides, particularly of Al, Fe, and Mn [22, 23], and to the clay content of sediments [5].

A significant aspect to be taken into account when As (V) adsorption is studied is the potential competition between arsenate and phosphate for surface sorption sites. Phosphate concentration has been considered a critical factor in the adsorption or release of As from solid phases [24]. Arsenate and phosphate behave both as oxyanions and present striking similarities such as quasi-identical pK_a_ values and charged oxygen atoms [25]. Phosphate strongly competes with As (V) for surface sites, inhibiting As (V) adsorption by Fe and Al oxides [26]. In the literature, the mobilization of As by P from sediments has been widely reported by Kaplan and Knox, Bauer and Blodau, Stollenwerk et al., Rubinos et al., and Rubinos et al. [19, 27–30], amongst others.

The role of organisms that colonize the sediment water interface must also be taken into account. In recent years, several studies have treated the sorption and removal of arsenate by means of iron-oxidizing bacteria [31], the seaweed* Lessonia nigrescens *[32], and by sulphate-reducing bacteria [33]. Therefore, we hypothesize that As adsorption capacity may be affected by the presence of biofilms in the water-sediment interface. Costerton (2007) defines a biofilm as a universal community of microorganisms (bacteria, fungi, cyanobacteria, algae, and protozoa) linked to wet surfaces or interfaces and embedded by a polymeric matrix (EPS) which allows an efficient water, nutrients and gas exchange between constituent populations and the outside environment [34]. Biofilms play an important role in rivers systems as they constitute the interface between the overlying water and the sediments and are the first to interact with dissolved substances such as nutrients, organic matter, and toxicants [35]. 

The literature and investigations on the behaviour of epipsammic biofilms on the retention of heavy metals and metalloids are scarce, so as in natural river ecosystems such as at the microcosm and mesocosm scales. Published researches, as previously mentioned, are focused on As retention by sediments and by certain isolated organism but not on the whole river bed system with the presence of multispecies biofilms, which will be one of the objectives of this study.

In this work, the effect of epipsammic biofilms developed over riverbed sediments on As retention is evaluated as well as their environmental role in river systems with presence of problematic As (V) concentrations. The capacity of As (V) retention of biofilm-sediment systems will be compared to that of the sediment without biofilm, as well as the potential remobilization produced after the retention. The effect of the biofilm on As retention in the presence of equimolar P concentrations was also assessed.

## 2. Materials and Methods

### 2.1. Sediment Sample

The sediment sample was obtained in the Anllóns River, in a noncontaminated area upstream of the town of Carballo. A complex sample was collected with a small plastic shovel from the top 5 cm at various points at the same site and taken to the laboratory in hermetic plastic containers topped up to prevent oxidation. The Anllóns basin is located in the NW of Spain and was selected because gold mining activities were carried out in the area during the Roman Empire and between 1895 and 1910 [36]. Arsenopyrite associated to Au produced elevated As concentrations in the bed sediments downstream the mineralized areas [17, 29, 30, 36]. Nowadays, the exploitation of the mineralized area is under study, causing social concern and controversy among the locals.

### 2.2. Sediment and River Water Characterization

Grain size distribution of the sediment was determined as it was described by Guitián and Carballas, and the fractions were classified as coarse sand (2–0.2 mm), fine sand (0.2–0.05 mm), coarse silt (0.05–0.02 mm), fine silt (0.02–0.002 mm), and clay (<0.002 mm) [37]. Total P (P_*T*_) was determined by acid digestion (HF, H_2_SO_4_, HCl, 10 : 1 : 10) followed by colorimetric determination with molybdenum blue, as described by Murphy and Riley [38].

Nitrogen was determined by wet digestion with H_2_SO_4_, by using the Kjeldahl method as described in Guitián and Carballas [37]. The concentration of total organic carbon (TOC) of the samples was determined according to the procedure proposed by Sauerlandt and modified by Guitián and Carballas [37], in an automatic titration system.

Sediment native As concentration was determined by X-ray fluorescence spectrometry (custom built, equipped with a Philips high-voltage generator and a Mo anade of 2.2 kW as X-ray source), following the method described by Devesa-Rey et al. [36]. The concentration of Al, Fe, and Mn was also determined.

River water was collected and filtered by 0.45 *μ*m to be employed as biofilm growth medium in the laboratory in order to better reproduce the natural conditions for biofilm growth. pH and conductivity were determined, as well as soluble P by means of an acid digestion with H_2_SO_4_ followed by colorimetric determination with ammonium molybdate [39].

### 2.3. Native Biofilm Growth

A natural biofilm was grown in indoor systems during 15 days over 8 g of riverbed sediment, using 60 mL of natural river water as nutrient supplier, in small plastic containers of 100 mL. The samples were subjected to day-night cycles (12 h of light with 3,109 lux of intensity) to reproduce approximately the natural environmental conditions. The overlying river water was replaced each 5 days together with the addition of 0.5 mL of inoculum (fresh river biofilm) in order to stimulate the biofilm growth. Once the biofilm was developed, the overlying water was removed, and its total P was measured by acid digestion with H_2_SO_4_. 

### 2.4. Arsenate Sorption Experiments

To evaluate the sorption capacity and desorption behaviour of the biofilm-sediment system, *batch* experiments were conducted with 8 g sediment and their corresponding formed biofilm. In parallel, samples without biofilm following the same treatment of the biofilm-sediment samples were used as controls.

60 mL of As (V) solutions with initial concentrations (*C*_0_) of 0, 5, 25, 50, 100, 250, and 500 *μ*g L^−1^, prepared in 0.01 M CaCl_2_ solutions as background electrolyte, were added to the systems. All the experiments were carried out in triplicate. Arsenate solutions were prepared from a stock standard solution of 1000 mg L^−1^ (Panreac, Barcelona, Spain). All the samples were prepared in triplicate. The batch experiments were carried out at room temperature (20 ± 2°C). Eh and pH measurements were carried out with a Thermo Scientific Orion Dual Star meter with a combined Redox/ORP electrode and with a AQUAPRO pH electrode (Beverly, USA), respectively. After 24 h, a pseudoequilibrium state was reached, and the overlying water was taken (pipetting without altering the system). Aliquots were filtered through a 0.45 *μ*m Whatman filter, and As concentration (*C*_*e*_) of the samples was determined by Inductively Coupled Plasma Spectrometry (ICP-MS, Varian 820 MS) with collision reaction interface (CRI) technology to reduce polyatomic interferences. The adsorbed As (V) concentrations (*Q*_ads_) for the sediment or biofilm-sediment systems were obtained by the difference between *C*_0_ and *C*_*e*_, taking into account the water volume and sediment weight.

For the study of the desorption behaviour, 60 mL of 0.01 M CaCl_2_ solutions were added to the previous loaded systems. After 24 h, aliquots of the overlying water were extracted by gently pipetting. Again, the samples were filtered and As concentration measured by ICP-MS. The weight of the samples was controlled in every moment to calculate the mass of As desorbed. All the experiments were carried out at pH 5.5 adjusted by addition of 0.1 M NaOH or HCl solutions.

### 2.5. Influence of Phosphorus Presence on Arsenate Sorption Process

To assess the influence of P presence on arsenate sorption, experiments with solutions of equimolar As(V): P concentrations were carried out, using the aforementioned procedure and concentrations used for the As (V) sorption experiments. P solutions were obtained by dissolution of KH_2_PO_4_ (Panreac, Barcelona, Spain). As and P concentrations in the supernatants were determined by ICP-MS. 

### 2.6. Sorption Modelling

The adsorption experimental data were fitted using a linear equation, four two-parameters models (Freundlich, Langmuir, Dubinin-Radushkevich, and Temkin), and three three-parameters models (Redlich-Peterson, Sips, and Toth). 

The linear equation was given by (1):

(1) $$\begin{array}{c} Q_{e}=AC_{e}-B, \end{array}$$

where *Q*_*e*_ is the adsorbed or desorbed As concentration for the sediment or biofilm-sediment system, *A* is the slope, and *B* is the content of native arsenic. 

The Freundlich equation (2) is used to describe heterogenous systems characterized by a heterogenous factor 1/*n*:

(2) $$\begin{array}{c} Q_{e}=K_{f}C_{e}^{1/n}, \end{array}$$

where *K*_*f*_ and *n* are empirical constants of the Freundlich model which are referred to as the capacity and intensity of adsorption, respectively [40].

The Langmuir equation (3) assumes monolayer coverage of adsorbate over a homogenous adsorbent surface:

(3) $$\begin{array}{c} Q_{e}=\frac{\left(Q_{\mathrm{Max}}bC_{e}\right)}{\left(1+bC_{e}\right)}, \end{array}$$

where *Q*_*Max*⁡_ is the maximum adsorption capacity of the system and b is a constant related to the energy bonds As-sediment and As-biofilm sediment interface [41].

The Dubinin-Radushkevich model isotherm is generally given by (4) [42]:

(4) $$\begin{array}{c} Q_{e}=q_{D}\exp\left(-B_{D}{\left[RT\ln\left(1+\frac{1}{C_{e}}\right)\right]}^{2}\;\right), \end{array}$$

where *B*_*D*_ is related to the mean free energy of sorption per gram of the sorbate as it is transferred to the surface of the solid from infinite distance in the solution [43]. 

The Temkin isotherm model contains a factor which takes into the account of adsorbent-adsorbate interactions and has been generally used in the form of (5) [44]:

(5) $$\begin{array}{c} Q_{e}=\frac{RT}{b_{T}}\ln\left(A_{T}C_{e}\right). \end{array}$$

The Redlich-Peterson empirical equation (6) incorporates features of both Langmuir and Freundlich equations [45]. It can be applied to represent adsorption equilibrium over a wide concentration range:

(6) $$\begin{array}{c} Q_{e}=\frac{\left(K_{R}C_{e}\right)}{\left(1+a_{R}C_{e}^{\beta }\right)}. \end{array}$$

Sips model isotherm is also called Langmuir-Freundlich isotherm [46]. At low sorbate concentrations, it reduces to a Freundlich isotherm, and at high sorbate concentrations, a monolayer sorption capacity is predicted [47]:

(7) $$\begin{array}{c} Q_{e}=\frac{\left(K_{S}C_{e}^{1/b_{S}}\right)}{\left(1+a_{S}C_{e}^{1/b_{S}}\right)}. \end{array}$$

The Toth isotherm model is an empirical equation useful in describing heterogeneous adsorption systems [48]. Equation (8) exhibits the most general form of this model:

(8) $$\begin{array}{c} Q_{e}=\frac{\left(K_{t}C_{e}\right)}{\left[{\left(a_{t}+C_{e}\right)}^{1/t}\right]}. \end{array}$$

The parameters of all studied models were estimated by nonlinear regression procedure employing Table Curve software (Jandel Scientific).

### 2.7. Statistical Analyses

Five error functions were tested in order to choose the best model to fit the experimental data. These error functions were the coefficient of determination (*R* ^2^), sum of absolute errors (EABS), hybrid fractional error function (HYBRYD), average relative error (ARE), and Marquardt's percent standard deviation (MPSD) and were calculated employing the equations described by Foo and Hameed [49].

The adsorbed concentrations of the different studied systems were evaluated by one-factor analysis of variance (ANOVA). Critical *F* values (*α* = 0.05) were used to evaluate if the factor is significant. In the case of positive significance, post hoc analyses using the Duncan comparison test (*α* = 0.05) were performed to establish statistical differences between the means (SPSS 19.0 statistical package).

### 2.8. Theoretical Aqueous Speciation

Visual MINTEQ V 3.0 was employed to theoretically calculate As species in the solutions and to determine their saturation degree, expressed as saturation index (SI) with respect to mineral phases, by means of thermodynamic calculations and (9):

(9) $$\begin{array}{c} \text{SI}=\log\left(\frac{\text{IAP}}{K_{C}}\right), \end{array}$$

where IAP is the ionic activity product of the specific dissolution-precipitation reaction and *K*_*C*_ is equilibrium constant. Negative SI indicates a mineral which has potential to dissolve, whereas positive SI shows a mineral which has thermodynamic potential to precipitate [50].

To study the influence of the pH and organic matter in arsenic speciation, sweeps with values between 4 and 10 and 1 and 10 mg L^−1^, respectively, were performed. 

## 3. Results and Discussion

### 3.1. Sediment and River Water Characterization

The sediment sample collected for this study showed a predominance of the sandy fraction, with an average value of 86.3%, and only 6.7% of clayey fraction. Total organic matter content for the sediment was of 13.9 ± 0.6 g kg^−1^. P and N concentrations presented average values of 471.9 ± 43.7 and 629.6 ± 98.3 mg kg^−1^, respectively. The total As concentration of sediment determined by X-ray fluorescence spectrometry was 11.8 mg kg^−1^, whereas the sediment content of Al, Fe, and Mn was 50.4, 51.9, and 1.2 g kg^−1^, respectively.

The values of pH, electrical conductivity, and soluble P concentration in the river water were 6.87, 71.80 *μ*S cm^−1^, and 0.21 mg L^−1^, respectively.

### 3.2. Arsenate Retention

**Figure 1:**
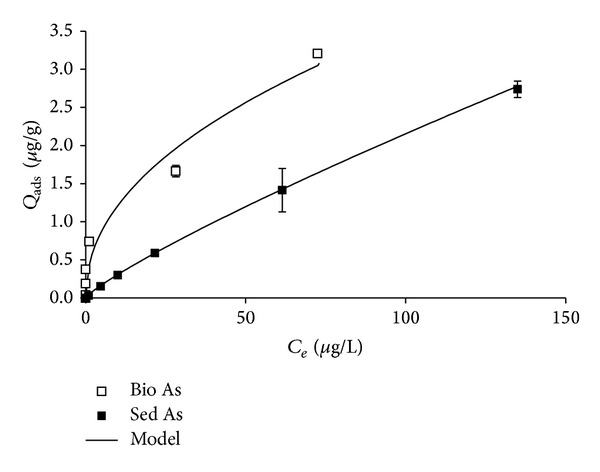
As (V) retention by biofilm-sediment and sediment systems in function of dissolved As equilibrium concentration.

Figure 1 shows the experimental data for As (V) sorption in function of the equilibrium As concentrations in the solution for the sediment and biofilm-sediment systems. *Q*_ads_ increased with increasing initial concentrations in all cases, thus indicating that the adsorption was not at its maximum. The values of *Q*_ads_ for sediment system without biofilm are in the range of the data reported by Stollenwerk et al. [19] and Borgnino et al. [20] but slightly lower because they used a different ratio (solution/sediment), lower native As concentrations in sediments, and higher added As concentrations.

It is noteworthy that *Q*_ads_ values for the samples with biofilm were higher than for the samples without biofilms. The average percentage of As adsorbed with respect to *C*_0_ for the sediment and biofilm-sediment system was 78.9 ± 3.5 and 96.9 ± 6.6%, respectively. The difference between *Q*_ads_ for the biofilm-sediment system and for the sediment without biofilm increased in the range of studied concentrations from 6.81 × 10^−3^ up to 4.69 × 10^−1^ *μ*g g^−1^ and was significant from As solutions of concentrations ≥50 *μ*g L^−1^. This may be explained by an increase in the specific surface area and the number of sorption sites and functional groups due to the presence of the biofilm, as well as arsenate biouptake by microorganisms which constitute the biofilm. Arsenate could enter cells through phosphate-transporting systems [51]. Arsenate bioaccumulation and biouptake in green algae was studied by Karadjova et al. [52] and by Wang et al. [53]. Karadjova et al. [52] reported that intracellular As increased linearly when As (V) concentrations increased up to 50 *μ*M, followed by a single saturation plateau.

Studies about As retention onto episammic biofilm have not been reported. However, the removal of heavy metals by means of bacteria biofilm has been widely and successfully studied. For example, a biofilm of *Arthrobacter viscosus* was applied to remove Cr(VI), Cd(II), and Ni(II) [43, 54–56] whereas a biofilm of *Pseudomonas aeruginosa* was employed to the removal of Cr(III), Ni(II), and Co(II) [57]. 

These results highlight the important role that biofilms may play in river environments by increasing As (V) retention. Biofilms could promote As sequestration from the water column; therefore, they could be potentially employed as a bioremediation tool for contaminated waters due to the larger surface area of the biofilm, with more functional groups where As can be adsorbed.

**Figure 2:**
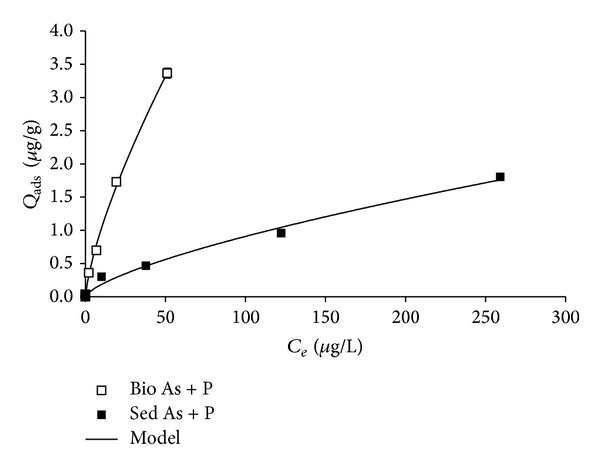
As (V) retention for biofilm-sediment and sediment systems in function of dissolved As equilibrium concentration in the presence of equimolar P concentrations.

Figure 2 presents *Q*_ads_ for As, in the presence of equimolar P for the sediment and biofilm-sediment systems. Again, *Q*_ads_ values for the biofilm-sediment system were higher than for sediment without biofilm. The difference between *Q*_ads_ for both systems increased in the range of studied concentrations from 0.00 up to 1.56 *μ*g g^−1^ and was significant again from an initial concentration of 50 *μ*g As L^−1^. At the highest As concentration, this difference was three times higher than in the experiments without P. This behaviour could be attributed to the P presence which caused a significant reduction on As (V) adsorbed by the sediment without biofilm (a 25% reduction at the highest added As concentrations), whereas no significant effect was detected in systems where biofilm took part. 

The competition between phosphate and arsenate for sorption sites in sediments has been widely reported. Thus, for example, Stollenwerk et al. [19] reported that for concentrations of As (V) of 100 *μ*g L^−1^, the presence of 2 mg L^−1^ of phosphate completely inhibited As (V) adsorption. Rubinos et al. [29] also showed that the addition of increasing concentrations of phosphate enhanced the As (V) release from sediments of the Anllóns River, and, in the same line, Rubinos et al. [30] confirmed that P increased the As mobilization from these sediments in a wide range of pH (3–10). Nevertheless, data have not been reported about the effect of P on As (V) sorption in the presence of biofilms. In this study, no significant effect of phosphate in As concentration of the overlying water was observed in systems with biofilm, which could be explained by the increase of sorption sites promoted by the biofilm and/or by the increase of intracellular arsenic uptake by microorganisms which constitute the biofilm.

Desorption processes were negligible at the lowest and middle concentrations in all the studied systems and represented less than 0.5% of the sorbed As concentrations at the highest initial As concentration. 

### 3.3. Sorption Modelling

Figures 1 and 2 showed the sorption curves for all the studied systems with their corresponding fits. Sorption isotherms were of type I according to Brunauer's classification [58].  Table 1 shows the parameters for the different tested models. According to them, sorption data were satisfactorily adjusted by all the models. The values of the parameter *A* of the linear model were higher for the biofilm-sediment systems, especially in the presence of phosphate, while the lowest was obtained for sediment without biofilm in the presence of P.

The equilibrium arsenic concentration (EAC) is defined analogously to equilibrium phosphorous concentration (EPC) as the concentration of As that is supported by the sediment when in contact with an ambient solution such that no arsenate is either gained or lost by the sediment [17]. When As concentrations in water are higher than EAC, the sediment would act as a sink for As, whereas for water As concentrations lower than EAC, the sediment would act as a source of As. Calculated EAC values ranged from 2.62 to 18.28 *μ*g L^−1^; the lowest EAC corresponded to the biofilm-sediment system with P and the highest to the sediment system with P, thus pointing to a higher risk of As transfer towards the water column.

Among the analysed two-parameters models, the Freundlich model was considered the most suitable to fit experimental data in all cases; the highest *R* ^2^ values and the lowest values of other error functions are shown (Table 2). The better fits of Freundlich model are indicative of the heterogeneous surface of the solid phases studied. Langmuir model also successfully adjusted the experimental data, with *R* ^2^ values above 0.92 in all cases, whereas the Dubinin-Radushkevich and Temkin models were not completely satisfactory.

The estimated maximum adsorption capacity of the sediment and biofilm-sediment systems, obtained from the Langmuir model, was 6.6 and 6.8 *μ*g g^−1^, respectively, and 4.5 and 7.8 *μ*g g^−1^, respectively, in the presence of P. These values fall within the range of, but slightly lower than, those reported by Stollenwerk et al. [19] when studying As adsorption oxidized aquifer sediments. Again, these results highlight the key effect that the presence of biofilm causes in the fate of As in the river system, mainly in the presence of P.

The essential characteristics of the Langmuir isotherm can be expressed in terms of an equilibrium parameter, *R*_*L*_, which allows to determine if the adsorption process is favourable or unfavourable [59]. Equation (10) shows the relationship between *R*_*L*_ and *C*_0_:

(10) $$\begin{array}{c} R_{L}=\frac{1}{\left(1+bC_{0}\right)}. \end{array}$$

**Figure 3:**
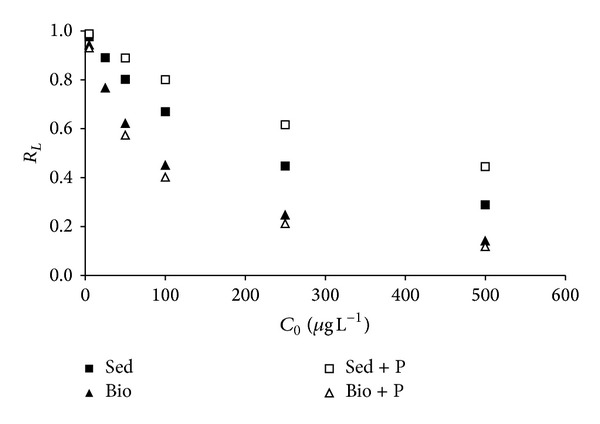
*R*_*L*_ values for all studied systems in function of initial As concentrations.

The values of *R*_*L*_ ranged between 0 and 1 for all the analyzed concentrations, which corresponds to a high affinity favorable adsorption process, being more favorable at the highest initial As concentrations. As it could be seen in Figure 3, the biofilm-sediment systems in the presence of phosphate present the highest affinity for As (the lowest *R*_*L*_), whereas the sediments in the presence of phosphate show the lowest one (the highest *R*_*L*_).

Gibbs free energy of adsorption process could be obtained from Langmuir and Temkin models by means of (11) and (12), respectively:

(11) $$\begin{array}{c} \ln\left(\frac{1}{b}\right)=\frac{{\Delta G}^{0}}{RT}, \end{array}$$

(12) $$\begin{array}{c} \ln\left(\frac{1}{A_{T}}\right)=\frac{{\Delta G}^{0}}{RT}. \end{array}$$

The values of Gibbs free energy yield negative values in all cases, which indicate that the adsorption process was always spontaneous. The biofilm-sediment system in the presence of P showed the most negative value by applying (11) (−34.50 kJ mol^−1^) and (12) (−43.10 kJ mol^−1^), whereas the sediment system with P presented the least negative value (−30.08 kJ mol^−1^ by (11) and −39.83 kJ mol^−1^ by (12)). The systems without P showed an intermediate behaviour, having more spontaneous As (V) retention in biofilm-sediment system. Therefore, the presence of biofilms jointly with P presence suggests that As retention process was more favoured.

Table 2 shows also that the three-parameters models, especially Sips and Toth models, also satisfactorily fit the experimental data based on the high *R* ^2^ values (>0.95) and low values of the error functions. 

### 3.4. Theoretical Aqueous Speciation

The calculations performed by Visual MINTEQ indicated that in the studied systems, arsenic was present as inorganic As (V), with approximately 95% of total species present as H_2_AsO_4_^−^. Negative values of SI were found in all studied cases, which indicated that the conditions are not favourable for the precipitation of As minerals. At pH 4, the monovalent H_2_AsO_4_^−^ species prevails (approximately 98% of total aqueous As), whereas at pH 10, the bivalent HAsO_4_^2−^ species prevails (approximately 96% of the total aqueous As). In the studied conditions, the calculations did not predict significant complexation of As with the dissolved organic matter.

## 4. Conclusions

The biofilm increases the As (V) sorption capacity of the studied sediment. An input of P, at equimolar P: As concentrations, reduces the sorption of As (V) on the sediment, whereas no significant effect is exhibited by systems with biofilm. The Freundlich model is the best, amongst the two-parameters models, to fit the As retention in these systems, which is indicative of their heterogeneous surface. The sorption of As is spontaneous and favourable in all cases, especially under the combined effect of biofilm and P, whereas the desorption this As retained is not significant. Overall, epipsammic biofilms play a key role in the fate and mobility of As in riverine environments and particularly in the transference of As from the water column to the sediment. They seem to enhance the sorption capacity and the affinity of the sediments for As, especially in environments where both As and P occur simultaneously.

## Figures and Tables

**Table 1:** Parameters of theAs(V)sorption models.

	Sediment	Biofilm	Sediment	Biofilm
	As(V)	As(V) + P	As(V)	As(V) + P
Linear				
*A *	2.02 × 10^−2^	6.60 × 10^−3^	4.15 × 10^−2^	6.51 × 10^−2^
*B *	7.19 × 10^−2^	1.21 × 10^−1^	2.83 × 10^−1^	1.71 × 10^−1^
Freundlich				
*k*_*f*_	4.35 × 10^−2^	3.71 × 10^−2^	4.12 × 10^−1^	1.91 × 10^−1^
*n *	1.18	1.44	2.14	1.37
Langmuir				
*Q*_*Max*⁡_	6.60	4.52	6.79	7.78
*b *	4.94 × 10^−3^	2.50 × 10^−3^	1.22 × 10^−2^	1.49 × 10^−2^
Dubinin-Radushkevich				
*q*_*D*_	3.23	1.54	3.614	3.72
*B*_*D*_	5.07 × 10^−4^	3.50 × 10^−4^	1.04 × 10^−4^	4.73 × 10^−5^
Temkin				
*A*_*T*_	2.48	1.28 × 10^−1^	2.94	4.79 × 10^−1^
*b*_*T*_	4.93 × 10^3^	5.68 × 10^3^	4.84 × 10^3^	2.66 × 10^3^
Redlich-Peterson				
*k*_*r*_	6.00 × 10^−2^	3.87 × 10^5^	1.25 × 10^6^	7.80 × 10^5^
*a*_*r*_	5.59 × 10^−1^	1.04 × 10^7^	3.11 × 10^6^	4.07 × 10^6^
*B *	2.56 × 10^−1^	3.05 × 10^−1^	5.32 × 10^−1^	2.70 × 10^−1^
Toth				
*k*_*t*_	4.69 × 10^−2^	5.32 × 10^−2^	2.59 × 10^−1^	1.97 × 10^−1^
*a*_*t*_	3.91	6.02 × 10^−3^	3.62 × 10^−2^	3.60 × 10^−1^
*t *	5.89	2.68	2.43	3.62
Sips				
*k*_*s*_	4.09 × 10^−2^	1.04 × 10^−1^	1.07 × 10^−2^	3.45 × 10^−2^
*a*_*s*_	1.15	4.28	330.52	66.13
*b*_*s*_	7.18 × 10^−4^	−2.15 × 10^−1^	−9.84 × 10^−1^	−9.32 × 10^−1^

**Table 2:** Values of error functions for each model and for each analysed system.

	Sediment		Native biofilm	
	As(V)	As(V) + P	As(V)	As(V) + P
Freundlich				
*R* ^2^	0.999	0.990	0.953	0.998
EABS	7.83 × 10^−2^	2.89 × 10^−1^	1.36	2.26 × 10^−1^
HYBRYD	8.31	3.77 × 10^−1^	7.29 × 10^1^	3.13 × 10^1^
ARE	5.94	2.51 × 10^1^	5.21 × 10^1^	2.09 × 10^1^
MPSD	1.40 × 10^2^	5.37 × 10^2^	8.00 × 10^2^	5.06 × 10^2^
Langmuir				
*R* ^2^	0.996	0.977	0.927	0.998
EABS	2.85 × 10^−1^	4.31 × 10^−1^	1.34	2.09 × 10^−1^
HYBRYD	6.01	4.78 × 10^1^	7.86 × 10^1^	3.49 × 10^1^
ARE	4.29	3.19 × 10^1^	5.61 × 10^1^	2.33 × 10^1^
MPSD	6.18 × 10^1^	5.98 × 10^2^	8.68 × 10^2^	5.30 × 10^2^
Dubinin-Rabushkevich				
*R* ^2^	0.925	0.854	0.912	0.930
EABS	1.24 × 10^1^	3.15	4.87	8.65
HYBRYD	1.73 × 10^3^	1.79 × 10^2^	9.36 × 10^1^	3.68 × 10^2^
ARE	1.24 × 10^3^	1.19 × 10^2^	6.69 × 10^1^	2.46 × 10^2^
MPSD	2.51 × 10^4^	2.37 × 10^3^	1.60 × 10^3^	5.10 × 10^3^
Temkin				
*R* ^2^	0.760	0.839	0.810	0.895
EABS	5.48	9.30 × 10^−1^	1.19	1.51
HYBRYD	5.41 × 10^2^	3.77 × 10^1^	1.37 × 10^1^	4.80 × 10^1^
ARE	3.86 × 10^2^	2.52 × 10^1^	9.79	3.20 × 10^1^
MPSD	6.74 × 10^3^	4.20 × 10^2^	1.91 × 10^2^	5.98 × 10^2^
Redlich-Peterson				
*R* ^2^	0.999	0.990	0.953	0.998
EABS	1.74 × 10^−2^	2.44 × 10^−1^	1.40	1.97 × 10^−1^
HYBRYD	3.46	1.25 × 10^1^	7.31 × 10^1^	6.36
ARE	2.47	8.35	5.22 × 10^1^	4.24
MPSD	6.70 × 10^1^	1.96 × 10^2^	8.02 × 10^2^	7.87 × 10^1^
Toth				
*R* ^2^	0.999	0.987	0.948	0.998
EABS	1.06 × 10^−2^	3.60 × 10^−1^	1.27	2.39 × 10^−1^
HYBRYD	1.57	3.81 × 10^1^	7.50 × 10^1^	3.18 × 10^1^
ARE	1.12	2.54 × 10^1^	5.35 × 10^1^	2.12 × 10^1^
MPSD	2.83 × 10^1^	5.22 × 10^2^	8.26 × 10^2^	5.07 × 10^2^
Sips				
*R* ^2^	0.999	0.999	0.984	0.978
EABS	1.12 × 10^−2^	3.34 × 10^−1^	0.99	2.69 × 10^−1^
HYBRYD	1.62	3.68 × 10^1^	6.80 × 10^1^	3.30 × 10^1^
ARE	1.23	2.27 × 10^1^	4.97 × 10^1^	2.31 × 10^1^
MPSD	2.89 × 10^1^	5.01 × 10^2^	7.98 × 10^2^	5.29 × 10^2^

## Nomenclature

*a*_*R*_: Redlich-Peterson model parameter (6) (L *μ*g^−1^)
*a*_*S*_: Sips model parameter (7) (L *μ*g^−1^)
*a*_*t*_: Toth model parameter (8) (*μ*g g^−1^)
*A*: Linear model parameter (1) (L g^−1^)
*A*_*T*_: Temkin isotherm equilibrium binding constant (L *μ*g^−1^) (5)
*b*: Langmuir model parameter (3) (L *μ*g^−1^)
*b*_*S*_: Sips model parameter (7) (−)
*b*_*T*_: Temkin model parameter (5)
*B*: Linear model parameter (1) (*μ*g g^−1^)
*B*_*D*_: Dubinin-Radushkevich parameter (4)
*C*_*e*_: Solution pseudoequilibrium concentration (mg L^−1^)
*C*_0_: Solution initial concentration (*μ*g L^−1^)
*k*_*f*_: Freundlich model parameter (2) (L g^−1^)
*K*_*S*_: Sips model parameter (7) (L *μ*g^−1^)
*K*_*R*_: Redlich-Peterson model parameter (6) (L *μ*g^−1^)
*K*_*t*_: Toth model parameter (8) (L *μ*g^−1^)
*n*: Freundlich model parameter (2) (−)
*q*_*D*_: Dubinin-Radushkevich model parameter (4) (*μ*g g^−1^)
*Q*_ads_: Adsorbed concentration by sediment or biofilm-sediment interface (*μ*g g^−1^)
*Q*_*des*⁡_: Desorbed concentration by sediment or biofilm-sediment interface (*μ*g g^−1^)
*Q*_*e*_: Solid-phase equilibrium concentration (*μ*g g^−1^)
*Q*_*Max*⁡_: Maximum adsorption capacity (*μ*g g^−1^) (3)
*R*_*L*_: Langmuir equilibrium parameter (10) (−)
*t*: Toth model parameter (8) (−)
*T*: Absolute temperature (K) ((4), (5), (11), and (12))
*R*: Gas constant (J mol^−1^ K^−1^) ((4), (5), (11), and (12)).

### Greek Letters

*β*: Redlich-Peterson parameter (6) (−).
